# Autologous bone marrow-derived mesenchymal stem cells for interstitial fibrosis and tubular atrophy: a pilot study

**DOI:** 10.1080/0886022X.2021.1968432

**Published:** 2021-09-07

**Authors:** Lei Zhang, Xingqiang Lai, Yuhe Guo, Junjie Ma, Jiali Fang, Guanghui Li, Lu Xu, Wei Yin, Zheng Chen

**Affiliations:** Department of Organ Transplantation, The Second Affiliated Hospital of Guangzhou Medical University/The Second Clinical Medicine School of Guangzhou Medical University, Guangzhou, China

**Keywords:** Interstitial fibrosis and tubular atrophy, mesenchymal stem cells, chronic allograft nephropathy, renal function

## Abstract

**Background:**

Mesenchymal stem cells (MSCs)-based therapy has shown promising results for renal injury. In this study, the efficacy and safety of autologous bone marrow-derived mesenchymal stem cells (BM-MSCs) in treating nonspecific interstitial fibrosis and tubular atrophy (IFTA) were evaluated.

**Methods:**

From March 2011 to January 2013, 11 renal transplanted patients with IFTA were recruited. At baseline, patients were given one intra-arterial infusion of BM-MSCs; 7 days and 1 month later, another two intravenous infusions of cells were followed. Serum creatinine, creatinine clearance rate, and serum cystatin-C at baseline and 7 days, 1 month, 3 months, 6 months, and 12 months after the intra-arterial infusion of BM-MSCs were used to assess renal function. At baseline and 6 months, histological examination based on hematoxylin-eosin, Masson’s trichrome and periodic acid-Schiff staining and immunohistochemistry for transforming growth factor β1 (TGF-β1) and connective tissue growth factor (CTGF) was performed. Adverse events were recorded to evaluate the safety of BM-MSCs treatment.

**Results:**

At 12 months, the renal function of 6 patients (54.5%) was improved, 3 (27.3%) were stable and 2 (18.2%) were worsened. At 6 months, the mean IFTA scores of all participators were similar with the baseline (1.73 ± 0.41 vs.1.50 ± 0.0.77, *p* = 0.242); however, it was significantly decreased when only 6 patients with improved renal function were analyzed (1.67 ± 0.41 vs. 1.08 ± 0.20, *p* = 0.013). Besides, decreased expression of TGF-β1 and CTGF were also observed at 6 months. During 1 year follow-up period, only two minor complications including infection and allergy were observed.

**Conclusion:**

Our results demonstrated that autologous BM-MSCs are safe and beneficial for IFTA patients. **Abbreviations**: MSCs: mesenchymal stem cells; BM-MSCs: marrow-derived mesenchymal stem cells; IFTA: interstitial fibrosis and tubular atrophy; CAN: chronic allograft nephropathy; CNIs: calcineurin inhibitors; Scr: serum creatinine; CCr: creatinine clearance rate; Cys-C: cystatin-C; TGF-β1: transforming growth factor β1; CTGF: connective tissue growth factor

## Introduction

The introduction of calcineurin inhibitors (CNIs), including cyclosporine and tacrolimus, has revolutionized the field of kidney transplantation by improving renal graft’s short-term outcomes; however, the long-term survival of grafts is yet unsettled [1]. CNI nephrotoxicity was quite common in kidney transplant recipients, which was considered as an important non-immunological cause of interstitial fibrosis and tubular atrophy (IFTA). IFTA, used named chronic allograft nephropathy (CAN), is one of the main causes of late graft loss [2]. Nowadays, there is no curative treatment for IFTA. In clinical practice, strategies such as CNI reduction [3] or conversion from CNI to other immunosuppressive drugs such as sirolimus [4] and mycophenolate mofetil (MMF) [5] have been tested for treating CAN (i.e., IFTA). Some pieces of evidence indicated that conversion from CNI to sirolimus or MMF is associated with a short-term improvement of renal function. However, the adverse effects of these drugs limit their clinical use. In a systematic review, authors evaluated twelve randomized controlled trials that focused on the immunosuppressive strategies of CAN and chronic allograft dysfunction (CAD). They found conversion from CNI to sirolimus or MMF appears to be beneficial for some patients. Both drugs are associated with adverse events that caused medication discontinuation in up to 10.5% of patients taking sirolimus and 24% of patients taking MMF. Therefore, exploring other strategies with better efficacy and/or less adverse effects is of great importance [6].

Mesenchymal stem cells (MSCs) are a type of cells with characteristics of self-renewal, multi-directional differentiation potentials, and low immunogenicity [7]. MSCs have revealed beneficial effects on promoting renal repair due to their immunosuppressive, immunomodulatory, and reparative properties, which makes them an attractive option for potential use in renal transplantation [8–10]. In addition, MSCs also showed some potential in treating IFTA. For example, in an animal study, Franquesa and colleagues compared the treatment effects of bone marrow mononuclear cells (BMCs) and MSCs and found a reduction of interstitial fibrosis and tubular atrophy in MSCs treated CAN rats [11].

In this study, we tried to investigate the 1-year outcome of autologous bone marrow-derived MSCs (BM-MSCs) in treating renal transplanted patients with IFTA. We anticipate this strategy may bring a potential benefit to IFTA patients.

## Materials and methods

### Patients

From March 2011 to January 2013, renal transplanted patients with biopsy-proven IFTA in the Second Affiliated Hospital of Guangzhou Medical University were considered for enrollment in this pilot study. All participators met the following inclusion criteria: (1) with biopsy-proven IFTA based on the Banff 2007 criteria; (2) in treatment with cyclosporine/tacrolimus based therapy; (3) had stable serum creatinine level within 6 months before enrollment; (4) panel-reactive antibody (PRA) < 10%; (5) initial transplant, donors were younger than 60 years old and ABO compatible. Patients were excluded if (1) be complicated by biopsy-proven acute rejection; (2) be hepatitis B/C virus infector or carrier; (3) has a new tumor. This study was performed in accordance with the Declaration of Helsinki and approved by the Ethical Committee of the Second Affiliated Hospital of Guangzhou Medical University (ethics approval number: 201108). Written informed consent was obtained from all participators.

### BM-MSCs isolation, cultivation, and characterization

BM-MSCs isolation, cultivation, and characterization were performed according to the methods previously reported [10]. Briefly, approximately 50 mL of bone marrow was obtained by bone marrow puncture from recruited patients themselves and diluted in 100 mL of PBS. Under the instructions of manufacturers, mononuclear cells were collected using a lymphocyte separation medium (Sigma-Aldrich, St. Louis, MO, USA) and washed with PBS. The supernatants were removed; after then, Low glucose Dulbecco’s Modified Eagle’s medium (Hyclone Laboratories, Inc., Logan, UT, USA), changed once every 3 days, was used to culture cells in a 37 °C incubator. At 80–90% confluence, cells were dissociated with 0.25% trypsin and sub-cultured at a ratio of 1:2 until 10 passages and cryopreserved for further processing. Morphology observation, flowcytometric analysis of surface markers including CD44, CD29, CD105, CD48, and CD34, adipogenic and osteogenic differentiation tests were performed to characterize BM-MSCs.

### Treatment

**Figure 1.**
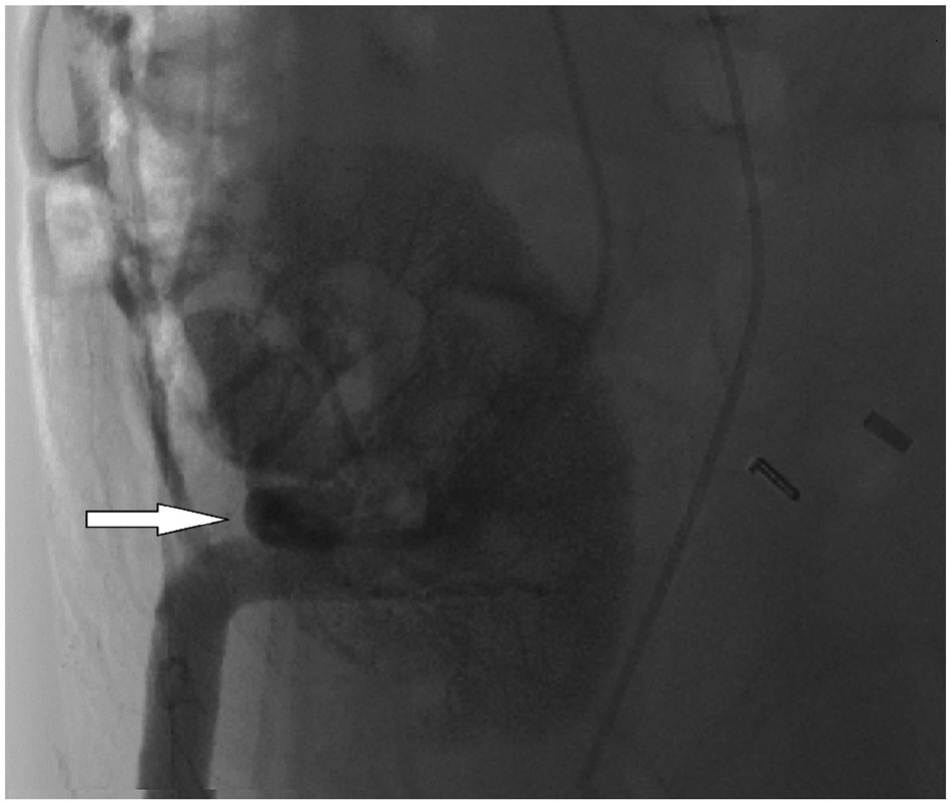
Representative DSA angiography photograph of kidney during intra-arterial infusion of BM-MSCs. White arrow indicates transplanted BM-MSCs. DSA: digital subtraction angiography; BM-MSCs: bone marrow-derived mesenchymal stem cells.

After characterization, cryopreserved BM-MSCs were thawed and tested negative for endotoxin, HIV, syphilis, hepatitis B/C virus, bacteria fungus, and mycoplasma before infusion. BM-MSCs were given in three times, at baseline (day 0), 7 days, and 1 month later. Intra-arterial infusion of BM-MSCs was performed on day 0. Briefly, under general anesthesia, the femoral artery was punctured by the Seldinger technique using a 4.0 F Cobra catheter (Terumo Co., Tokyo, Japan). 7 mL iohexol were injected at a speed of 2 mL/s for digital subtraction angiography (DSA). 5 × 10^6^ BM-MSCs, diluted in 20 mL saline, were then quickly injected into the artery (Figure 1). Later, a pressure bandage was applied at the puncture site for 4 h. 7 days and 1 month later, the 2nd and 3rd intravenous infusion of BM-MSCs, diluted in 50 mL saline, were given to patients within 20 min at a dose of 1 × 10^6^ cells per kilogram of body weight.

### Renal function evaluation

Serum creatinine (Scr), creatinine clearance rate (CCr), and serum Cystatin-C (Cys-C) at baseline and various time points after the BM-MSCs arterial infusion (7 days, 1 month, 3 months, 6 months, and 12 months) were used to assess renal function. Notably, at baseline, 7 days, and 1 month time points, samples were collected prior to BM-MSCs infusion to obtain Scr, CCr, and Cys-C. 12 months after the intra-arterial infusion of BM-MSCs, renal function was assessed: improved when Scr was decreased by 10% or CCr increased by 10%, worsened when Scr was increased by 10% or CCr decreased by 10%, and stable when Scr or CCr was changed by less than 10%.

### Histological examination

Renal specimens, obtained by needle-core biopsies (16 gauge) that were performed under ultrasonographic guidance at baseline and 6 months after the intra-arterial infusion of BM-MSCs, were fixed in 4% formaldehyde, paraffin-embedded, and then processed for routine hematoxylin-eosin (H&E), Masson’s trichrome and periodic acid-Schiff (PAS) staining. Two experienced pathologists were invited to evaluate these histological figures in a blinded manner according to the Banff 2007 [12] and Banff 2017 guidelines [13].

### Immunohistochemistry

Immunohistochemical evaluation of the protein expression of transforming growth factor β1 (TGF-β1) and connective tissue growth factor (CTGF) in all patients was carried out at baseline and 6 months after the intra-arterial infusion of BM-MSCs. Briefly, 4 μm sections of paraffin-embedded renal biopsy tissue were deparaffinized and hydrated through graded alcohol series. The endogenous peroxidase activity was quenched by incubation in 3% H_2_O_2_ for 15 min. After blocking with 2% normal serum at room temperature for 20 min, the slides were incubated overnight at 4 °C with the following primary antibodies: anti-rabbit TGF-β1 antibody (1:100, Beijing Bioss biological Technology Co., Ltd, Beijing, China); anti-rabbit CTGF antibody (1:100, Beijing Bioss biological Technology Co., Ltd, Beijing, China). After being washed with PBS, sections were then incubated with goat anti-rabbit biotinylated secondary antibody (Santa Cruz, CA, USA), visualized by 3,3′-diaminobenzidine (DAB), counterstained with hematoxylin, and mounted with resin. Photographs were then taken using a Leica microscope. The integral optical density (IOD) values of figures were measured using the JD-801 pathological imaging analysis system (Jiangsu JEDA Science-Technology Development Co., Ltd., Jiangsu, China).

### Adverse events

During the study period, treatment-related adverse events such as bleeding, allergy, infection, new tumor formation, renal arterial embolization, and pseudoaneurysm, if presented, were recorded and treated as per the demand.

### Statistical analysis

Statistical analysis was performed using SPSS 19.0 (SPSS, Inc., Chicago, IL, USA). Continuous covariates were analyzed using paired *t*-test. A *p-*value of <0.05 was considered significant.

## Results

### Baseline characteristics of all participators

In total, 11 renal transplanted patients with IFTA, including 7 males and 4 females, were included in this study. Their average age was 39.1 ± 13.7 years and the average time post-transplant was 5.4 ± 3.9 years. Baseline characteristics of all participators were presented in Table 1.

**Table 1.** Baseline characteristics of all participants (*n* = 11).

Characteristics	Values
Age, years	39.1 ± 13.7
Gender, Male/Female	7/4
Time post-transplant, years	5.4 ± 3.9
Delayed graft function (%)	18.2
Cold ischemia time, h	6.2 ± 1.7
HLA mismatches	3.5 ± 1.6
CNI drug, tacrolimus/cyclosporine	7/4
Primary kidney diseases	
Chronic glomerulonephritis	9
IgA nephropathy	1
Polycystic kidney	1

### MSCs characterization

**Figure 2.**
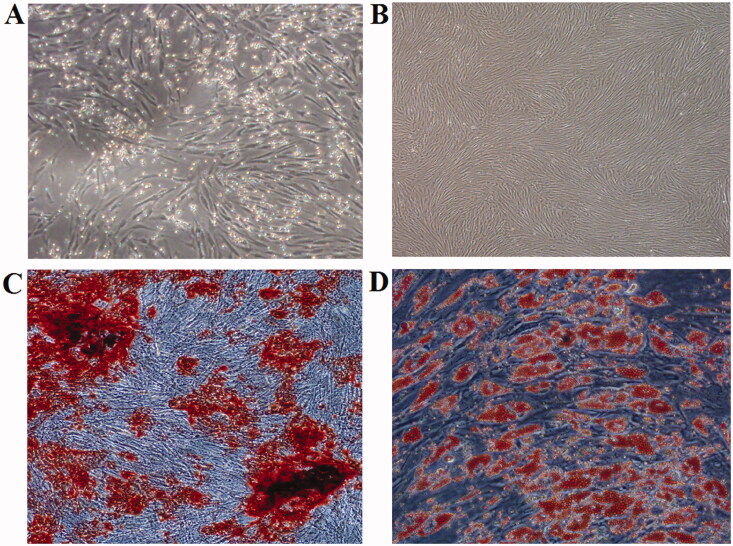
Characterization of human BM-MSCs expanded *in vitro*. (A) Cultured cells with spindle-shape, fibroblastic-like morphology at 7 days of culture; (B) cultured cells grew well with typical spindle-shape (at 10 passages); (C) osteogenic differentiation, Alizarin red staining; (D) adipogenic induction, Oil Red O staining.

After 7 days of culture, adherent cells with spindle-shaped fibroblastic-like morphology were observed and cell colonies were formed (Figure 2(A)). At 10 passages, cultured cells grew well with a typical spindle-shape (Figure 2(B)). 14 days after osteogenesis induction, nodous cells together with lots of calcium salt deposition were observed after alizarin red staining (Figure 2(C)). 14 days after adipogenic induction, lipid vacuoles in cells were visualized by Oil Red O staining (Figure 2(D)). Flow cytometry results confirmed that cultured cells in our experiment were expressed as CD44^+^CD29^+^CD105^+^CD48^−^CD34^−^ (Figure not shown).

### Renal function before and after BM-MSCs treatment

**Figure 3.**
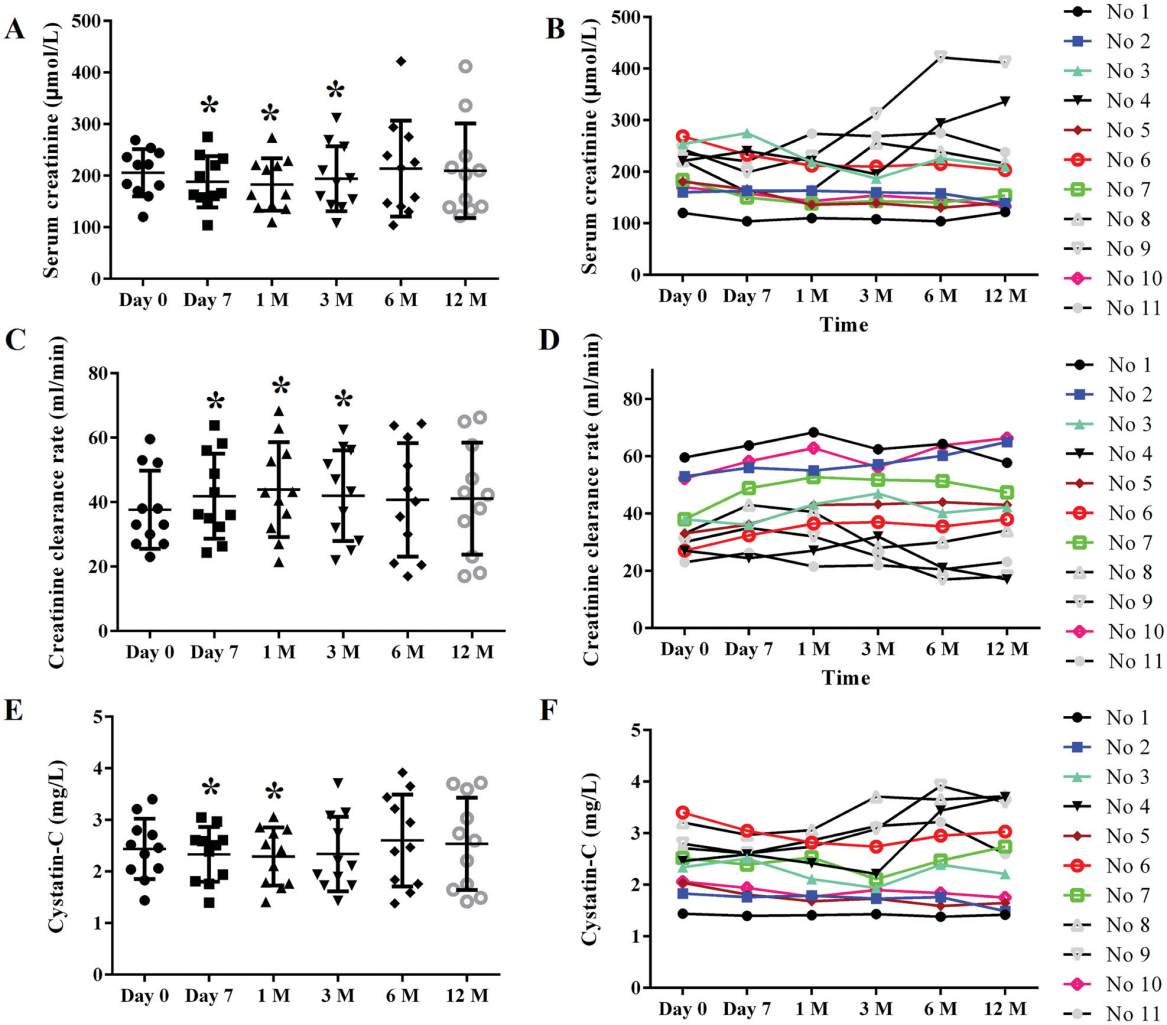
Renal function evaluated by Scr, CCr and Cys-C. (A) Scatter diagram of Scr of all participators at different time points after BM-MSCs infusion; (B) line chart of individual Scr; (C) Scatter diagram of CCr of all participators at different time points after BM-MSCs infusion; (D) line chart of individual CCr; (E) Scatter diagram of Cys-C of all participators at different time points after BM-MSCs infusion; (F) line chart of individual Cys-C. Scr: serum creatinine; CCr: creatinine clearance rate; Cys-C: Cystatin-C. * *p* < 0.05, vs. baseline.

Our results showed that Scr levels (μmol/L) of patients at baseline, 7 days, 1 month, 3 months, 6 months, and 12 months after the intra-arterial infusion of BM-MSCs were 205.5 ± 45.8, 188.1 ± 49.7, 182.6 ± 50.9, 193.9 ± 62.8, 213.6 ± 93.0, and 209.4 ± 91.8, respectively. As compared with the baseline, Scr was significantly decreased at 7 days, 1 month, and 3 months (*p* < 0.05) but kept unchanged at other time points (*p* > 0.05) (Figure 3(A)). The CCr levels (mL/min) of patients at baseline, 7 days, 1 month, 3 months, 6 months, and 12 months were 37.6 ± 12.1, 41.8 ± 13.2, 43.9 ± 14.7, 42.0 ± 14.1, 40.7 ± 17.6, and 41.1 ± 17.4, respectively. CCr was significantly increased at 7 days, 1 month, and 3 months as compared with that of the baseline (*p* < 0.05). CCr at other time points was on an uptrend but did not show a significant difference as compared with the baseline (*p* > 0.05) (Figure 3(C)). Cys-C showed a similar change trend as Scr. The Cys-C level (mg/L) of patients at baseline, 7 days, 1 month, 3 months, 6 months, and 12 months was 2.44 ± 0.58, 2.33 ± 0.53, 2.29 ± 0.56, 2.34 ± 0.72, 2.60 ± 0.89, and 2.54 ± 0.90, respectively. When compared with the baseline, Cys-C was significantly decreased at 7 days and 1 month (*p* < 0.05) (Figure 3(E)).

To better know the Scr, CCr, and Cys-C changes of the individual patient, line charts of Scr (Figure 3(B)), CCr (Figure 3(D)), and Cys-C (Figure 3(F)) were also presented. Based on changes of Scr and CCr, the renal function of 6 patients (54.5%) was assessed as improved, 3 (27.3%) as stable, and 2 (18.2%) as worsened at 12 months after the intra-arterial infusion of BM-MSCs.

### Histological improvement was noticed after BM-MSCs treatment

**Figure 4.**
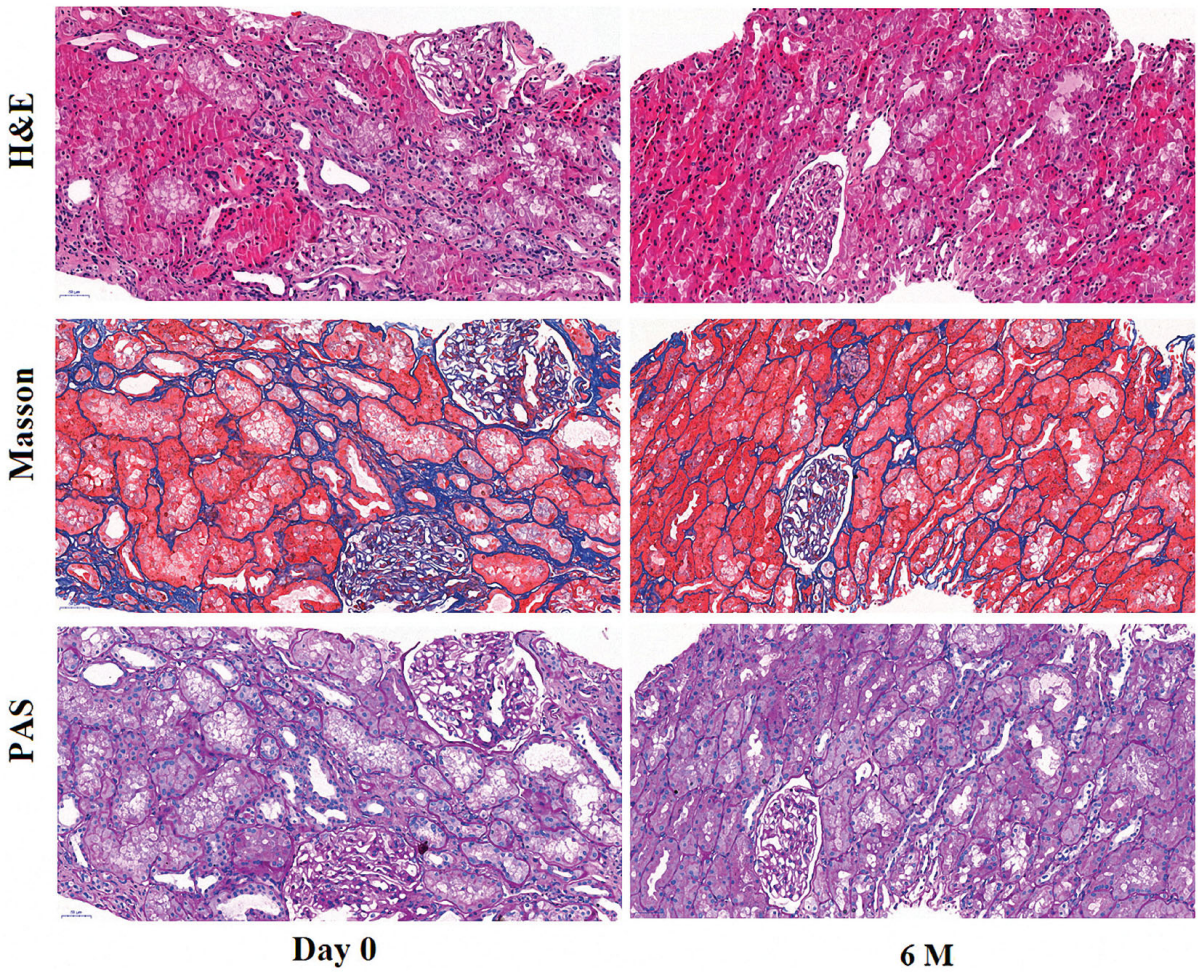
Representative histopathology figures before and after BM-MSCs treatment. At day 0, typical IFTA histopathology characteristics such as interstitial fibrosis, inflammatory cells infiltration, tubular epithelial atrophy and glomerulosclerosis were noticed; these characteristics were somewhat alleviated at 6 months after the intra-arterial infusion of BM-MSCs. BM-MSCs: bone marrow-derived mesenchymal stem cells.

At baseline, typical histopathology characteristics such as interstitial fibrosis, inflammatory cell infiltration, tubular epithelial atrophy, and glomerulosclerosis were observed; 6 months after the intra-arterial infusion of BM-MSCs, the degree of these histopathology characteristics were somewhat alleviated (Figure 4). On the whole, the mean IFTA score of this time point was on a downtrend but did not show a significant difference as compared with that of the baseline (1.73 ± 0.41 vs.1.50 ± 0.0.77, *p* = 0.242). Interestingly, the mean IFTA score was significantly decreased when only six patients with final improved renal function were analyzed (1.67 ± 0.41 vs. 1.08 ± 0.20, *p* = 0.013).

### Protein expression of TGF-β1 and CTGF was decreased after MSCs treatment

**Figure 5.**
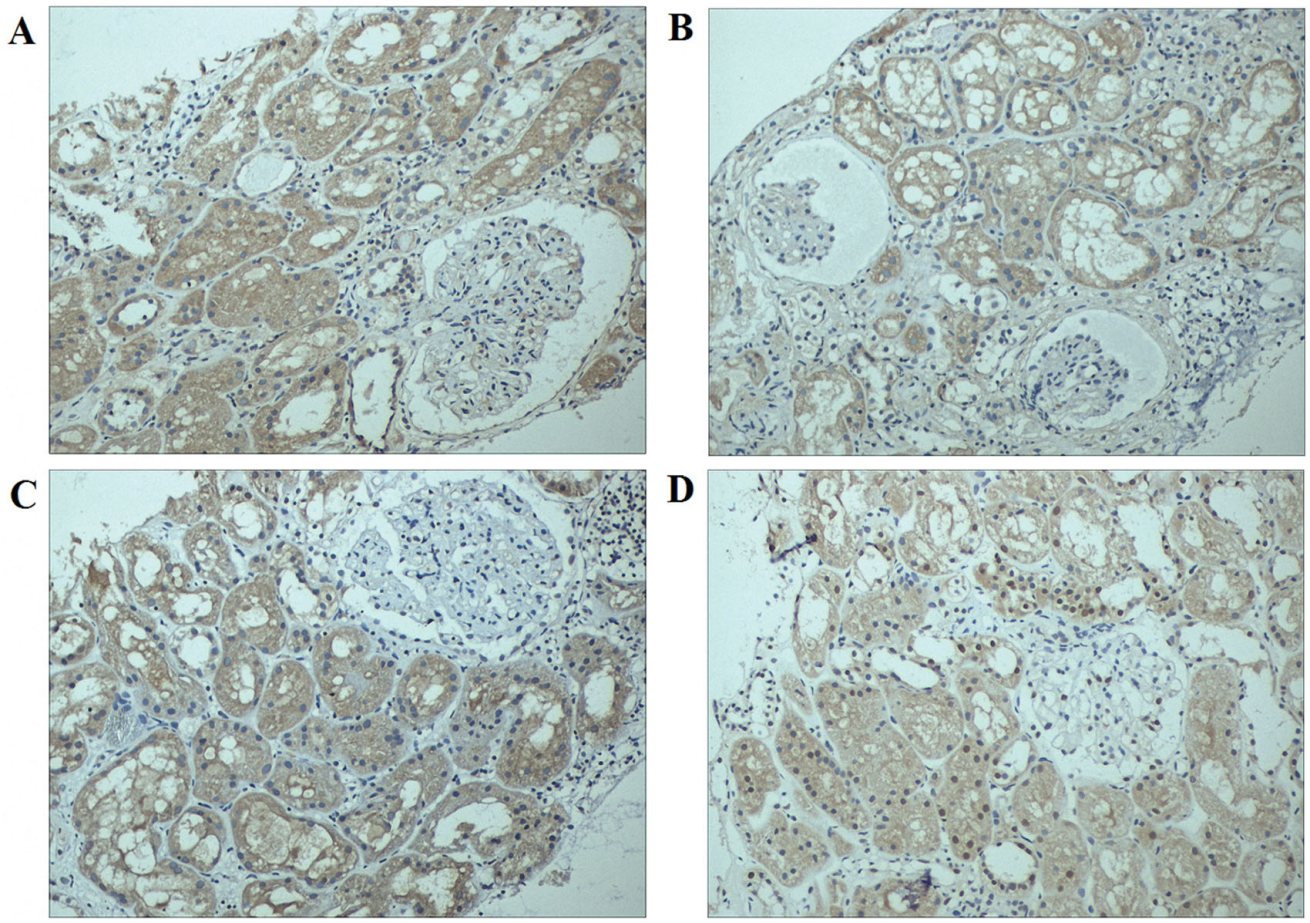
Representative immunohistochemistry figures of TGF-β1 and CTGF before and after BM-MSCs treatment. (A) TGF-β1 at baseline; (B) TGF-β1 at 6 months; (C) CTGF at baseline; (D) CTGF at 6 months. The protein expressions of TGF-β1 and CTGF were significantly decreased at 6 months after the intra-arterial infusion of BM-MSCs as compared with baseline. TGF-β1: transforming growth factor β1; CTGF: connective tissue growth factor; BM-MSCs: bone marrow-derived mesenchymal stem cells.

TGF-β1 and CTGF are two important renal fibrotic factors. As shown in Figure 5, the protein expressions of TGF-β1 (0.247 ± 0.052 vs. 0.196 ± 0.064) and CTGF (0.242 ± 0.044 vs. 0.204 ± 0.070) were significantly decreased at 6 months after the intra-arterial infusion of BM-MSCs as compared with baseline (*p* < 0.05).

### Adverse events

During 1 year follow-up period, infection symptoms such as fever, cough, and expectoration were noticed in one patient at 3 days after the intra-arterial injection of BM-MSCs. Allergy symptoms such as urticaria and pruritus all over the body were observed in another patient during the second intravenous infusion of BM-MSCs. The two were recovered after symptomatic treatment. No episodes of bleeding, new tumor formation, renal arterial embolization, and pseudoaneurysm were observed in all 11 patients.

## Discussion

MSCs represent a unique cell population with the ability of renewal and reconstruction and healing of injured tissues. Apart from bone marrow, MSCs can also be obtained from other tissues such as adipose tissue, umbilical cord, and peripheral blood. Bone marrow is the main source of MSC cells [14]. Therefore, in this pilot study, autologous bone marrow-derived MSCs were used to explore the efficacy and safety of MSCs in treating IFTA. During 1 year of observation, only two minor complications related to BM-MSCs treatment were observed. As for efficacy, our results showed that, 6 months after BM-MSCs treatment, the histopathology characteristics of patients, including IF and TA, were somewhat alleviated. At 12 months, the renal function of 6 patients was improved, 3 were stable, only 2 were worsened. These outcomes indicate that autologous BM-MSCs treatment for IFTA patients is safe and beneficial.

Nowadays, many clinical and experimental studies have demonstrated the therapeutic effects of MSCs in the early stage of kidney transplantation [8,10,15–17]; on the contrary, only a few studies have reported the relationship between MSCs and IFTA (or CAN). For instance, Reinders et al. evaluated the safety and feasibility of two intravenous infusions (1 × 10^6^ cells/kg) of autologous BM-MSCs in 6 patients with subclinical rejection (SCR) and/or IFTA. They found MSCs infusions were well-tolerated. In two patients, IFTA was alleviated after MSCs treatment [9]. In another clinical study, Ansary et al. used allogeneic MSCs to treat 10 CAN patients and found the mean serum creatinine was decreased and the mean creatinine clearance level was increased at 1, 3, and 6 months after MSCs infusion [18]. In an animal study performed by Franquesa et al., authors found that one single MSCs injection can preserve the renal function of CAN rats at 24 weeks after transplantation; besides, alleviation in interstitial fibrosis and tubular atrophy, less infiltration of T cells and macrophages, decreased expression of inflammatory cytokines while increased expression of anti-inflammatory factors was also observed in MSCs treated CAN rats [11]. In this study, we noticed that the renal function of the majority of patients was improved or stable during the 1-year follow-up period; in addition, histopathology characteristics of these patients with improved renal functions were alleviated at 6 months after the intra-arterial infusion of BM-MSCs. However, the Scr of two patients (#4 & #9) was significantly increased 3 and 1 month after the first intra-arterial infusion of BM-MSCs, respectively. We did not know the exact reason behind this phenomenon because we did not perform mechanism investigations. We suspect that the serious baseline conditions of these two patients may partly contribute to this outcome. In the future, appropriately designed studies are warranted to look for the applicable population of BM-MSCs.

As we know, the administration route is a key parameter that affects the migration and final effects of transplanted cells. In published studies, intravenous (IV) infusion was frequently used because it is a safe and easy strategy to deliver MSCs; however, one major concern about this administration route is that a majority of transplanted cells are trapped by organs such as lung, liver, and spleen. On the other hand, intra-arterial delivery of MSCs is safe and allows more cells to reach the target organ, making it an attractive route to deliver cells [19]. In a previous study, the implementation of one arterial injection (5 × 10^6^ cells) plus one intravenous infusion of MSCs (2 × 10^6^ cells/kg) has been proven to be safe in six *de novo* living-related kidney transplant recipients [20]. In the present study, one intra-arterial infusion of BM-MSCs (5 × 10^6^ cells) was firstly given under the guidance of DSA to insure more cells were delivered to the damaged kidney; 7 days and 1 month later, another two intravenous infusions of cells (1 × 10^6^ cells/kg) were followed to strengthen the therapeutic effects of BM-MSCs. We noticed that the Scr, CCr, or Cys-C levels of at least 8 patients were improved at day 7, indicates that intra-arterial infusion of autologous BM-MSCs has very positive therapeutic effects on the renal function of IFTA patients. On the contrary, the renal functions of the majority of IFTA patients were stable, even worsened, from day 7 to 12 months, suggests that the therapeutic effects of intravenous infusions of autologous BM-MSCs may be weaker than that of intra-arterial infusion.

TGF-β1, a critical profibrotic factor in the kidney, has been associated with the promotion of renal allograft interstitial fibrosis and thereby CAN [21]. CTGF is an important downstream mediator of TGF-β1 and mediates many actions of TGF-β1 in several fibrosis diseases [22,23]. The previous study has shown that both TGF-β1 and CTGF were over-expressed in the IFTA patients [24]. The expression of TGF-β1 and CTGF may be used to predict renal interstitial fibrosis [25,26]. In our study, the protein expressions of TGF-β1 and CTGF were decreased after BM-MSCs treatment, indicating BM-MSCs can alleviate the fibrosis degree of the kidney, which was in line with our histological observation.

As mentioned above, MSCs have exhibited some potential for treating IFTA; however, the exact mechanism of this action is still largely unclear. The following mechanisms may be involved in this process. Firstly, transplanted MSCs may differentiate into renal parenchymal cells such as glomerular endothelial cells, glomerular mesangial cells, and tubular epithelial cells to repair the damage of the kidney [27]. For instance, Li et al. reported that human adipose-derived MSCs can differentiate into renal tubular epithelium at an early stage of ischemia–reperfusion injury in the C57BL/6 mouse model. The dead cells were replaced by the differentiated donor cells, which contributed to the maintenance of structural integrity and subsequent tissue repair process [28]. However, the low engraftment rate of MSCs into the damaged areas seems to be inadequate to explain their therapeutic benefit. Secondly, MSCs can promote renal repair through their paracrine effects. In previous reports, MSCs have shown anti-renal fibrosis effects through secretion of various growth factors and cytokines such as epithelial growth factor (EGF), fibroblast growth factor (FGF), hepatocyte growth factor (HGF), vascular endothelial growth factor (VEGF), insulin-like growth factors-1 (IGF-1) and transforming growth factor-beta (TGFβ) [29,30]. These factors secreted by MSCs have trophic, immunomodulatory, antiapoptotic, and proangiogenic properties and thus be beneficial for renal repair. Growing pieces of evidence have suggested that the repair effects of MSCs probably mainly depended on the paracrine mechanism, rather than engraftment and differentiation within the target organ [31,32]. For instance, Franquesa et al. used one single MSCs injection to treat CAN rats and could not found GFP^+^ labeled MSCs in parenchyma either early after cell infusion at 12 weeks or lately at 24 weeks. They concluded that the therapeutic effect of MSCs on IFTA seems to attribute to the immunomodulatory properties of MSCs [11]. In the present study, Scr, CCr, and Cys-C of majority patients were significantly improved shortly after intra-arterial infusion of BM-MSCs; while, BM-MSCs did not work out satisfactorily 3 months after infusion. Based on this observation, we suspect that the therapeutic effects of BM-MSCs on IFTA patients probably mainly depended on their paracrine effects.

The short-term effect of BM-MSCs in treating or preventing the progress of IFTA is promising; however, the long-term effects of these cells are not clear. In the present study, 3-year and 5-year follow-ups were also performed by our nurses to know the status of patients, emphasized on their renal function and whether occurrence of tumor. As expected, the renal function of patients significantly deteriorated with mean Scr (μmol/L) of 294.3 ± 211.7 at 3-year (one lost follow-up and two death; among 8 followed patients, 2 had dialysis) and 337.9 ± 243.2 at 5-year (one lost follow-up and three death; among 7 followed patients, 3 had dialysis) time point. No tumor event was reported in all followed patients. However, due to a long time of follow-up interval and indeterminate influence of other interventions, we did not present these results in the main text of this paper.

This study has some limitations. Firstly, in our study, the renal functions of 6 out of 11 IFTA patients were improved at 12 months; while, due to the small number of participators, we could not conclude which parts of patients were benefits from BM-MSCs treatment; Secondly, this study is a self-control study; if we had set ‘control group’, in other words, patients treated with pure CNIs based therapy or BM-MSCs therapy, it would be much helpful to identify the effects of BM-MSCs on IFTA.

## Conclusions

Our results demonstrated that the strategy consists of one intra-arterial infusion and two intravenous infusions of autologous BM-MSCs is safe and beneficial for IFTA patients. In the future, more studies are warranted to further explore the optimal administration route, dose, and infusion times of cells to improve the long-term benefits of MSCs-based therapy.

## Ethical approval

This study was performed in accordance with the Declaration of Helsinki and approved by the Ethical Committee of the Second Affiliated Hospital of Guangzhou Medical University (ethics approval number: 201108). Written informed consent was obtained from all participators.

## Authors’ contributions

Conceptualization, L. Zh. and Zh. Ch.; methodology, X.Q. L., J.J.M., J.L.F., G.H.L, L.X. and W.Y.; formal analysis, X.Q. L. and J.J.M.; investigation, J.L.F., G.H.L, L.X., and W.Y.; data curation, J.L.F., and G.H.L; writing – original draft preparation, L. Zh.; writing – review and editing, Zh. Ch.; supervision, L. Zh. and Zh. Ch.; funding acquisition, Zh. Ch. All authors gave final approval to the manuscript.
